# Can GPT-4 be a viable alternative for discussing complex cases in digital oral radiology? A critical analysis

**DOI:** 10.17179/excli2023-6373

**Published:** 2023-08-01

**Authors:** Lucas Alves da Mota Santana, Lara Góis Floresta, Êmilly Victória Maciel Alves, Marcos Antônio Lima dos Santos, Breno Ferreira Barbosa, Sara Juliana de Abreu de Vasconcellos, Carolina Vieira Valadares

**Affiliations:** 1 Department of Dentistry, Federal University of Sergipe (UFS), Aracaju, SE, Brazil; 2 Department of Dentistry, Tiradentes University (UNIT), Aracaju, SE, Brazil; 3 Department of Stomatology, Faculty of Dentistry, University of Sao Paulo (USP), São Paulo, SP, Brazil

## ⁯⁯

Dental radiographs are an invaluable resource for an adequate diagnosis process in dentistry (Altoukhi et al., 2021[1]). Commonly, they are used to analyze the integrity of dental components and supporting anatomical structures (such as enamel, dentin and alveolar bone, respectively), surgical planning and rehabilitation, location of foreign bodies and identification of pathological process (Bilgir et al., 2021[3]; Fiorellini et al., 2021[6]). However, misdiagnosis cases or underreporting can occur and, as a result, negatively impact in the clinical management of patients. Alternatively, artificial intelligence (AI) resources have shown to be useful tools in overcoming these problems, favoring clinical decision-making (Moharrami et al., 2023[11]; Pauwels, 2021[13]). In this discussion, we explore the possibility of applying these AI models in digital oral radiology, particularly GPT-4, to reduce diagnostic failures by professionals and stimulate discussions among researchers. 

AI is a reality in the contemporary world, and its benefits in health field are unquestionable, including robotic surgery, storage and management of clinical patient data, personalized healthcare, teleconsultations, and remote monitoring (Bhandari et al., 2020[2]; Dave and Patel, 2023[4]). Among the newest revolutionary technologies is GPT-4, a software based on the operating model of ChatGPT. Released in March 2023 by OpenAI (https://openai.com/chatgpt), this chatbot can interpret both images and texts as inputs, generating detailed descriptions similar to human-like language. For that, this software uses databank storage on a computer, in addition to web data, for the elaboration of responses (Lecler et al., 2023[8]; Lyu et al., 2023[10]). Therefore, GPT-4 may be considered a viable alternative for discussing complex cases in oral radiology, with the potential to identify image patterns or nuances indistinguishable to the human eye.

In the medical field, several authors have analyzed the accuracy of this AI model, with some studies showing satisfactory results that can be applied to dentistry. In their observational study, Rao et al. (2023[14]) assessed the decision-making power of ChatGPT regarding breast cancer screening through image exams. Interestingly, the preliminary results obtained in this study, demonstrated that the software achieved a correctness percentage of 88.9 %. On another hand, AI has been employed in dentistry such as for diagnosing dental diseases trough panoramic radiographs, as reported by Zhu et al. (2023[16]). These authors mentioned that AI was precise in recognizing residual roots (p < 0.05) and significantly reducing the evaluation time (p < 0.001). 

It is recognized that several factors are associated with pitfalls in the process of radiographic interpretation in dentistry, including clinical experience, technical knowledge, case complexity, lack of careful analysis, emotional stress, and work overload (Hegde et al., 2023[7]; Murdoch et al., 2023[12]). Besides, some bone pathologies can share similar radiographical findings and, unfortunately, be misdiagnosed (DI Lauro et al., 2022[5]; Liu et al., 2021[9]). Probably, most of these negative outcomes may be minimized by employing new AI tools and models, including GPT-4. With advancement of digital radiology, the generated image is transferred to the monitor screen and instantly analyzed by professional (Yoon et al., 2018[15]). Occasionally, a combination with ChatGPT is possible once the installed software would use these images for verification and subsequent description in its output. 

Like other AI models, this chatbot shows some limitations, such as team training time and the risk of bias analyses (Dave and Patel, 2023[4]). Nevertheless, it is expected that ChatGPT will acquire new versions with an increasing amount of data and provide feedback capable of improving the system and its performance in storing information. Despite the controversies and speculations, the advantages of this technology are undeniable. As long as ethical principles are respected, it has the potential to add a series of benefits to patients, physicians, and healthcare units. 

Thus, combining between digital oral radiology and the different AI models represent the starting point for irrevocable progress in the field of radiology. In summary, these resources can contribute to the early diagnosis of malignant neoplasms, with greater precision in results, as well as increasing the efficiency of clinical interventions. 

## Conflict of interest

None to declare.
